# Association of feeding practices with growth in infants: a longitudinal observational study in a rural district of Pakistan

**DOI:** 10.1136/bmjph-2024-001204

**Published:** 2025-03-28

**Authors:** Waliyah Mughis, Sheraz Ahmed, Fayaz Umrani, Sadaf Jakhro, Khaliq Qureshi, Amnat Mangrio, Arjumand Rizvi, Asad Ali

**Affiliations:** 1 Community Health Sciences, The Aga Khan University, Karachi, Sindh, Pakistan; 2 Paediatrics, Aga Khan University, Karachi, Pakistan

**Keywords:** Nutrition Surveys, Population Surveillance, Sociodemographic Factors

## Abstract

**Background:**

Exclusive breastfeeding (EBF) is recommended for the first 6 months of an infant’s life, but barriers to EBF persist due to sociocultural, economic and health-related factors in resource-poor settings. This study examines the association between feeding practices and malnutrition in a birth cohort from a rural district of Pakistan.

**Methods:**

Data were collected from a cohort of n=2697 infants, up to 6 months of age, through routine household visits by community health workers. The study analysed demographic characteristics, feeding practices, anthropometric and health information of infants and their mothers.

**Results:**

Breastfeeding practices varied, with EBF being more common in the first month, while predominant breastfeeding (breastmilk with non-milk liquids) was most prevalent from 1 to 6 months. Almost all (98.3%) infants had been breastfed at some point between birth and 6 months but <10% were being exclusively breastfed by 6 months of age, with a significant proportion already identified as wasting (14.7%), stunted (36.7%) and underweight (38.5%) at baseline (birth). Early initiation of complementary feeding or breastmilk alternatives before 4 months was significantly associated with increased odds of wasting by 6 months of age (adjusted OR (AOR)=4.14; 95% CI: 1.95 to 8.77; p<0.001). Infants not born in medical facilities had higher risks of wasting (AOR=2.43; 95% CI: 1.13 to 5.21) and underweight status (AOR=1.74; 95% CI: 1.13 to 2.71).

**Conclusion:**

Our study indicates that infants were over four times more likely to be malnourished by 6 months if complementary feeding was initiated before 4 months of age. While causal relations/directionality cannot be established from these findings, we believe that parents may resort to suboptimal complementary feeding practices shortly after birth, due to perceived inadequate infant growth. A tailored approach addressing systemic barriers to optimal feeding practices is recommended for resource-constrained, nutrition-poor settings, such as rural Pakistan.

**WHAT IS ALREADY KNOWN ON THIS TOPIC**

- Mothers in resource-constrained rural areas of Pakistan face many barriers to exclusively breastfeeding their young infants under 6 months of age, leading to highly prevalent early initiation of complementary feeding.

**WHAT THIS STUDY ADDS**

- This study highlights the association between early initiation of complementary feeding and increased malnutrition risk. Infants started on complementary feeding before 4 months had a significantly higher risk of malnutrition/compromised growth (four times more likely) by 6 months of age. Non-facility births and delayed breastfeeding initiation were also associated with higher underweight and wasting risk.

**HOW THIS STUDY MAY AFFECT RESEARCH, PRACTICE OR POLICY**

- Further research is required to determine whether early initiation of complementary feeding causes poor growth, or if mothers of wasting infants notice that their child is not thriving and supplement breastfeeding/initiate complementary feeding earlier than recommended. Guidelines need to be developed on how to evaluate and feed infants who are failing to grow in the first 4 months despite attempted exclusive breastfeeding.

## Background

 While the WHO recommends exclusive breastfeeding (EBF) for the first 6 months of life to promote optimal growth and development,^1^ feeding practices for young infants in resource-constrained settings are affected by a complex interplay of sociocultural, economic, political (legislative, policy and state level) and health-related factors. Individual-level and systemic barriers to EBF in low- to middle-income countries, such as Pakistan, range from a lack of parental knowledge and education, limited social, economic, welfare and occupational support systems, financial constraints, cultural and social norms, maternity policies and maternal/child health issues.^2^,^6^ In the rural Pakistani context, these barriers include generally low awareness and cultural practices about prelacteal feeds, insufficient breastmilk production, undernutrition of mothers, abnormal breasts, maternal employment and occupation as fieldworkers, maternal and child ailments and influence of in-laws to start top-up feeds.^5 7 8^

To enable breastfeeding or other appropriate complementary feeding practices among mothers in resource-constrained settings, a multi-pronged approach involving education, health, social work, policy changes and community engagement is required^9^,^11^ to address systemic concerns beyond individual factors, such as maternal malnutrition, insufficient milk production and sociocultural practices. Even having knowledge about the benefits of EBF may not necessarily translate into appropriate EBF practices due to factors that families cannot address on the individual level.^12 13^ Affordable alternatives to breastmilk must be considered in cases where breastfeeding is truly not feasible or possible due to systemic and cultural factors, particularly for women in low-income settings who cannot breastfeed, cannot afford formula milk and do not have access to specialised lactation counsellors.^2 14^

Our research group conducted a community-based malnutrition intervention trial between 2016 and 2019 in which a birth cohort of moderately/severely malnourished and well-nourished children was compared on biomarkers of blood, urine and faecal samples at 3, 6 and 9 months of age.^15 16^ Feeding practices for infants of 6 months of age and below were also recorded. In the current study, we explored the birth cohort’s growth (wasting, stunting, underweight) and the associated parental choices for feeding practices in the first 6 months. We aimed to explore if infants are malnourished because they are not being exclusively breastfed in the first 6 months of life, or do mothers start early complementary feeding when they observe that EBF is insufficient for the child’s nutritional needs?

## Methods

### Study design

A longitudinal observational study was conducted in which quantitative data were collected on, and associations were analysed between infant anthropometric data (nutritional status) and feeding practices of a prospective cohort, from birth to 6 months of infant age.

### Setting and participants

This study was conducted in Matiari, a low-income rural district 185 km north of the city of Karachi, in Sindh province, Pakistan, comprising a population of 0.7 million. The Aga Khan University has established a human demographic surveillance system in Matiari wherein community health workers routinely conduct household visits to monitor and support maternal and child health; the demographic surveillance system (DSS) is also used for the identification and recruitment of participants for any research activities in the district. Monthly household surveillance for neonates was conducted via the DSS to follow this birth cohort for longitudinal growth analysis. In the current paper, we describe the feeding practices for and nutritional/growth data of this birth cohort of 2679 infants that was followed through routine household surveillance up to 6 months of age (March 2016 to November 2018).

### Patient and public involvement

Participants were not involved in the design, or conduct, or reporting, or dissemination plans of this research.

### Data collection tool

The survey comprised sections on demographic information of parents, child and household, IYCF (infant and young child feeding) practices, child illness and treatment history, anthropometric measurements of child, and maternal health and history.

Gestational age was determined using maternal recall and antenatal records where available. Feeding practices were defined as per the WHO’s IYCF guidelines,^17^ wherein *exclusive breastfeeding* refers to only giving an infant breastmilk for the first 6 months of life (no other food or water) and *predominant breastfeeding* refers to breastfeeding as well as the use of liquids (water and water-based drinks, fruit juice) ritual fluids and oral rehydration therapy drops or syrups (vitamins, minerals and medicines, excluding animal and formula milk). *Breastfeeding with other milk* refers to the use of formula and other animal milk (cow, goat, camel), and *no breastfeeding* refers to the use of all liquids other than breastmilk for feeding the infant. For infant anthropometry, the nutritional status of the child was classified as malnourished/wasted if weight-for-length z-score (WLZ) was <−2SD, stunted if length-for-age z-score was <−2SD and underweight if weight-for-age z-score (WAZ) was <−2SD.^18^

### Data collection procedure

The birth cohort of over 2600 children from the DSS was followed on a monthly basis up to 6 months of age by study data collectors. With the informed written or verbal consent of the mother (primary respondent), infants were registered in the DSS registry at birth. The data collectors administered the nutritional status and feeding practices survey with the mother and completed anthropometric measurements of the infant during seven household visits for the first 6 months of the infant’s life (birth visit+monthly visits up to 6 months of age).

### Analysis

We summarised categorical variables as frequencies and percentages. Normally distributed continuous variables were reported as mean and SD, while non-normally distributed continuous variables were reported as medians and IQRs. For univariate analysis, the χ^2^ test for categorical or dichotomous variables and the independent sample t-test for continuous variables were applied. Multivariate logistic regression was performed to determine the impact of several factors including feeding practices on the development of wasting. Effect estimates were reported as ORs with corresponding 95% CIs, and statistical significance was held at two-sided p<0.05 and p<0.10 for borderline significance. WLZ was computed using the WHO calculator which by default does not calculate z-scores of cases where birth length is <45 cm, and flags values where WLZ <−5SD or WLZ >5SD. Hence, birth length <45 cm and flagged values were excluded from the analysis. Since wasting (WHZ/WLZ) is the ratio of weight and height/length, chronically malnourished children with low length may not appear to be wasted even when their weight is low, which is why we present both underweight (WAZ) and wasting (WLZ) status of the cohort. Bivariate analysis was performed to identify the independent effect of each predictor on outcomes. The analysis was further adjusted for factors including child gender, place of birth, gestational age at birth, initiation of breastfeeding, duration of EBF and age of initiation of complementary diet. The analyses were conducted in STATA V.18.

## Results

The demographic characteristics and nutrition status at birth of n=2679 infants followed in routine household surveillance are summarised in table 1. Growth data were available for 1937 infants by 3 months of age, and 1275 infants by 6 months of age. The loss to follow-up over time was primarily due to the migration of participating families to neighbouring villages or towns not followed in the routine demographic surveillance. The median infant age at the time of enrolment was 8 days (IQR 4–12), and 91.6% of the infants were born at term. Most of the infants (58.4%) had breastfeeding initiated between 7 and 12 hours of birth, while only 236 (9.4%) infants had breastfeeding initiated within the first hour of birth. 98.3% of the infants had ever been breastfed at any point at the time of enrolment into the study. Infant anthropometry revealed that 14.7% of the infants were wasted, 36.7% were stunted and 38.5% were underweight at the time of enrolment (within a week of birth). The mean weight of the infants was 2.7 kg (SD±0.5) and mean length at the time of enrolment was 47.6 cm (SD±2.7). By 6 months of age, for n=1275 infants, mean weight was 6.34±0.94 kg and mean length was 62.97±2.7 cm.

**Table 1:** Baseline characteristics of participants at enrolment (at birth±1 week) (n=2679)

Characteristics		n
Age in days (mean±SD)	8.0±7.4	2679
Child gender		2679
Male	1375 (51.3%)	
Female	1304 (48.7%)	
Facility births	2184 (82.7%)	2641
Gestational age at birth (weeks)		2644
Preterm (GA <37 weeks)	223 (8.4%)	
Term (GA ≥37 weeks)	2421 (91.6%)	
Time of initiation of BF		2516
Within 1 hour	236 (9.4%)	
Between 1 and 6 hours	1469 (58.4%)	
Between 7 and 12 hours	350 (13.9%)	
>12 hours	461 (18.3%)	
Ever breastfed	2501 (98.3%)	2544
At birth		
Weight, kg (mean±SD)	2.7±0.5	2637
Length, cm (mean±SD)	47.6±2.7	2658
Weight for length z-score (mean±SD)	−0.9±1.1	2285
Length for age z-score (mean±SD)	−1.7±1.2	2658
Weight for age z-score (mean±SD)	−1.7±1.1	2637
Malnutrition status (%)		
Wasted (WLZ <−2SD)	336 (14.7%)	2285
Stunted (LAZ <−2SD)	976 (36.7%)	2659
Underweight (WAZ <−2SD)	1016 (38.5%)	2637
At 6 months		
Weight, kg (mean±SD)	6.34±0.94	1275
Length, cm (mean±SD)	62.97±2.7	1275

BF, breastfeeding; GA, gestational age; LAZ, length-for-age z-score; WAZ, weight-for-age z-score; WLZ, weight-for-length z-score.

**Figure 1:**
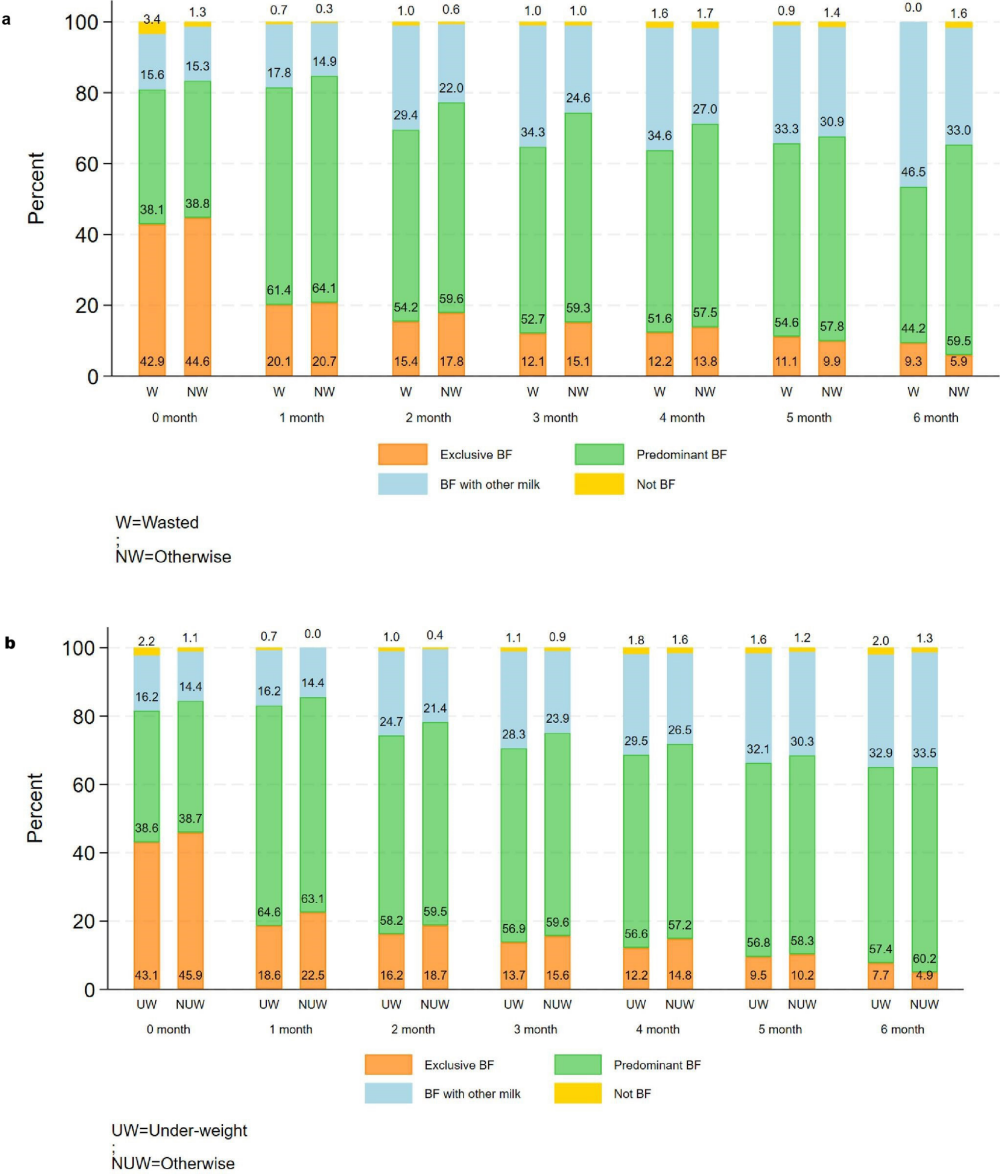
Comparison of breastfeeding practices by age (in months) among (**a**) wasted and (**b**) underweight children. *Predominant breastfeeding includes exclusive breastfeeding, breastfeeding plus water, and breastfeeding plus non-milk liquids/juice. BF with other milk includes breastmilk with formula milk or animal milk. BF, breastfeeding.

The prevalence of breastfeeding and complementary feeding practices across the first 6 months in wasting and non-wasting infants is summarised in figure 1a. While EBF was more common in the first month, complementary feeding and the introduction of other milk increased from 2 months of age, particularly in the wasted infants. Figure 1b depicts the breastfeeding practices of underweight and not-underweight children from birth to 6 months. The proportion of underweight children who were not breastfed at all was higher from birth and increased with age, compared with not-underweight children at 6 months. Additionally, underweight children were more likely to be introduced to complementary feeding right from the first month. The prevalence of this practice increased with age in both groups but remained higher among underweight children at 5 months of age.

In table 2, the comparative analysis of infant feeding practices against malnutrition status, specifically focusing on wasting and underweight conditions, revealed that the average age at which wasting developed was 3.7±0.9 months. Notably, infants who were wasted started other milk at a significantly younger age (1.9±1.6 months) compared with non-wasted infants (2.3±2.0 months, p=0.003), and also began complementary feeding earlier (4.3±1.2 months vs 5.3±1.2 months, p<0.001). EBF at 3 months was lower in the wasted group (10.7%) compared with the not-wasted group (14.0%, p=0.018). A similar trend was observed in underweight infants, where the initiation of other milk and complementary feeding occurred earlier (2.1±1.9 and 5.1±1.3 months, respectively) than in not-underweight infants (2.4±2.0 and 5.3±1.2 months, p=0.019 for both). Furthermore, at 6 months, EBF rates were lower in the underweight group (8.5% vs 5.3%, p=0.024) compared with the not-wasted infants.

**Table 2:** Comparison of infant feeding practices and malnutrition status at 3 and 6 months

Variable	Mean/frequency		Significance
Wasting	Wasted	Not wasted	P value
n	365	2314	
Age at development of wasting (mean±SD)	3.7±0.9	–	–
Duration of exclusively breastfeeding in months (mean±SD)*	0.3±0.8	0.4±0.9	0.25
Age at initiation of any other milk in months (mean±SD)	1.9±1.6	2.3±2.0	0.003
Age at initiation of complementary feeding in months (mean±SD)	4.3±1.2	5.3±1.2	<0.001
At 3 months			
n	280	1657	0.018
Exclusive breastfeeding	30 (10.7%)	232 (14.0%)	
Predominant BF	152 (54.3%)	990 (59.8%)	
Breastfed and other milk	94 (33.6%)	420 (25.4%)	
Not breastfed	4 (1.4%)	14 (0.8%)	
At 6 months			
n	30	1245	0.19
Exclusive breastfeeding	4 (13.3%)	78 (6.3%)	
Predominant BF	13 (43.3%)	732 (59.3%)	
Breastfed and other milk	13 (43.3%)	409 (33.1%)	
Not breastfed	0 (0.0%)	16 (1.3%)	

Underweight	Underweight	Not underweight	P value
n	1214	1439	
Age at development of underweight (mean±SD)	3.9±1.0	–	–
Duration of exclusively breastfeeding in months (mean±SD)*	0.3±0.8	0.4±1.0	0.035
Age at initiation of any other milk in months (mean±SD)	2.1±1.9	2.4±2.0	0.019
Age at initiation of complementary feeding in months (mean±SD)	5.1±1.3	5.3±1.2	0.019
At 3 months			
n	854	1071	0.028
Exclusive breastfeeding	102 (11.9%)	160 (15.0%)	
Predominant BF	492 (57.6%)	644 (60.2%)	
Breastfed and other milk	251 (29.4%)	257 (24.0%)	
Not breastfed	9 (1.1%)	9 (0.8%)	
At 6 months			
n	485	787	0.024
Exclusive breastfeeding	41 (8.5%)	41 (5.3%)	
Predominant BF	276 (57.1%)	468 (60.1%)	
Breastfed and other milk	156 (32.3%)	264 (33.9%)	
Not breastfed	10 (2.1%)	6 (0.8%)	

* Child considered on exclusive breastfeeding if they are continuously on exclusive breastfeeding with no breaks observed during follow-ups.

BF, breastfeeding.

The results of the multivariate binomial logistic regression analysis, aimed at identifying factors influencing the likelihood of wasting and underweight status in infants, are presented in table 3. A critical finding was that early complementary feeding (before 4 months) significantly increased the odds of wasting in infants by four times (adjusted OR (AOR)=4.14; 95% CI: 1.95 to 8.77; p<0.001). Male infants had a higher unadjusted risk of wasting than females (OR=1.56; 95% CI: 1.25 to 1.96; p<0.0001), although this factor was excluded from the adjusted model. Additionally, infants not born in a medical facility were at higher risk of wasting (AOR=2.43; 95% CI: 1.13 to 5.21; p=0.023). While breastfeeding status increased unadjusted odds of wasting (OR=2.75; 95% CI: 1.42 to 5.33; p=0.003), it was not included in the final adjusted model. For underweight status, male infants had increased odds (AOR=1.64; 95% CI: 1.18 to 2.29; p=0.003), and non-facility births also raised underweight risk (AOR=1.74; 95% CI: 1.13 to 2.71; p=0.013). Early complementary feeding (before 4 months) nearly doubled the odds of underweight status (AOR=1.97; 95% CI: 1.20 to 3.22; p=0.007). Breastfeeding initiation within the first hour and between 1 and 6 hours post-birth reduced unadjusted odds of underweight (OR=0.62 and 0.73, respectively; p<0.01), although these factors were not part of the adjusted model.

**Table 3:** Binary logistical model for wasting and underweight children (OR, 95% CI)

Variables in model	Wasting				Underweight			
	Unadjusted OR (95% CI)	P value	Adjusted OR (95% CI)	P value	Unadjusted OR (95% CI)	P value	Adjusted OR (95% CI)	P value
Sex								
Male	1.56 (1.25, 1.96)	<0.0001	–	–	1.41 (1.21, 1.64)	<0.0001	1.64 (1.18, 2.29)	0.003
Female	Ref.		–	–	Ref.		–	–
Facility birth								
No	1.35 (1.03, 1.78)	0.032	2.43 (1.13, 5.21)	0.023	1.58 (1.29, 1.94)	<0.0001	1.74 (1.13, 2.71)	0.013
Yes	Ref.		Ref.		Ref.		Ref.	
Ever breastfed								
No	2.75 (1.42, 5.33)	0.003	–	–	1.92 (1.03, 3.61)	0.041	–	–
Yes	Ref.		–	–	Ref.		–	–
Time of initiation of BF								
Within 1 hour	1.06 (0.68, 1.64)	0.8	–	–	0.62 (0.45, 0.86)	0.004	–	–
Between 1 and 6 hours	0.92 (0.68, 1.24)	0.566	–	–	0.73 (0.59, 0.90)	0.003	–	–
Between 7 and 12 hours	0.91 (0.61, 1.36)	0.654	–	–	0.80 (0.60, 1.05)	0.109	–	–
>12 hours	Ref.		–	–	Ref.		–	–
Gestational age at birth (weeks)								
Preterm (GA <37 weeks)	1.28 (0.89, 1.86)	0.189	–	–	1.34 (1.01, 1.78)	0.04	1.66 (0.93, 2.95)	0.087
Term (GA ≥37 weeks)	Ref.		–	–	Ref.		Ref.	–
Age at initiation of complementary feeding in months								
<4	4.28 (2.04, 8.96)	<0.001	4.14 (1.95, 8.77)	<0.001	1.98 (1.23, 3.21)	0.005	1.97 (1.20, 3.22)	0.007
>4	Ref.		Ref.		Ref.		Ref.	
Duration of EBF* (months)	0.92 (0.81, 1.06)	0.248	–	–	0.91 (0.84, 0.99)	0.036	–	–
Age at initiation of any other milk (months)	0.88 (0.81, 0.96)	0.003	–	–	0.93 (0.88, 0.99)	0.019	–	–

* EBF criteria met when child is continuously exclusively breastfeeding with no breaks observed during follow-ups.

BF, breastfeeding; EBF, exclusive breastfeeding; GA, gestational age.

Survival analysis for time to development of malnourished status (table 4) showed that early complementary feeding (before 4 months) significantly increased the risk of wasting, with an adjusted HR (AHR) of 4.59 (95% CI: 2.26 to 9.32; p<0.0001). Male infants had higher unadjusted risks of both wasting (HR=1.56; 95% CI: 1.25 to 1.93; p<0.0001) and underweight status (HR=1.59; 95% CI: 1.28 to 1.97; p<0.0001). Infants not born in a medical facility had increased adjusted risks of wasting (AHR=2.36; 95% CI: 1.15 to 4.86; p=0.019) and underweight (AHR=2.71; 95% CI: 1.04 to 7.08; p=0.042) status. Non-breastfed infants showed a higher unadjusted HR for wasting (HR=2.95; 95% CI: 1.70 to 5.14; p<0.0001) and underweight (HR=2.43; 95% CI: 1.33 to 4.43; p=0.004) status.

**Table 4:** Survival analysis for time to development of wasting and underweight status (HR, 95% CI)

Variables in model	Wasting				Underweight			
	Unadjusted HR (95% CI)	P value	Adjusted HR (95% CI)	P value	Unadjusted HR (95% CI)	P value	Adjusted HR (95% CI)	P value
Sex								
Male	1.56 (1.25, 1.93)	<0.0001	–	–	1.59 (1.28, 1.97)	<0.0001	–	–
Female	Ref.				Ref.			
Facility birth								
No	1.41 (1.09, 1.82)	0.008	2.36 (1.15, 4.86)	0.019	1.49 (1.16, 1.93)	0.002	2.71 (1.04, 7.08)	0.042
Yes	Ref.		Ref.		Ref.		Ref.	
Ever breastfed								
No	2.95 (1.70, 5.14)	<0.0001	–	–	2.43 (1.33, 4.43)	0.004	9.46 (1.20, 74.86)	0.033
Yes	Ref.						Ref.	
Time of BF Initiation								
Within 1 hour	1.04 (0.68, 1.57)	0.872	–	–	0.97 (0.65, 1.46)	0.898	–	–
1–6 hours	0.94 (0.7, 1.24)	0.647	–	–	0.87 (0.66, 1.15)	0.328	–	–
7–12 hours	0.89 (0.6, 1.31)	0.55	–	–	0.90 (0.61, 1.31)	0.568	–	–
>12 hours	Ref.				Ref.			
Gestational age at birth (weeks)								
Preterm	1.31 (0.93, 1.85)	0.118	–	–	1.23 (0.86, 1.77)	0.261	–	–
Term	Ref.				Ref.			
Age at initiation of CF (months)								
<4	4.89 (2.41, 9.90)	<0.0001	4.59 (2.26, 9.32)	<0.0001	4.02 (1.91, 8.46)	<0.0001	2.72 (1.04, 7.11)	0.042
>4	Ref.		Ref.		Ref.		Ref.	
Duration of EBF (months)*	0.90 (0.79, 1.03)	0.111	–	–	0.91 (0.80, 1.03)	0.138	–	–
Age at initiation of any other milk in months	0.86 (0.79, 0.93)	<0.0001	–	–	0.86 (0.80, 0.94)	<0.0001	–	–

* EBF criteria met when child is continuously exclusively breastfeeding with no breaks observed during follow-ups.

BF, breastfeeding; CF, complementary feeding; EBF, exclusive breastfeeding.

The associations between EBF, predominant BF, along with use of formula, animal milk and both types of non-breastmilk are summarised in online supplemental table 1, indicating that mixed feeding (breastfeeding combined with formula or animal milk) is associated with higher malnutrition rates compared with exclusive or predominant breastfeeding in infants at both 3 and 6 months.

## Discussion

The current study’s findings provide insights into the association between the initiation of complementary feeding and the likelihood of wasting among infants. While our study found that initiation of complementary feeding before 4 months of infant age was significantly associated with wasting by 6 months of age, it is imperative to note that this is not necessarily a causal relationship. Despite initially attempting EBF, parents may resort to complementary feeding practices when they perceive that the infant is not thriving with EBF by 4 months of age, which is why they may initiate complementary feeding early on. The inaccessibility to formula milk is well-documented not only in low-income but also in high-income settings.^19 20^ This underscores the inadequate implementation of standardised feeding practices, often leaving caregivers with little choice but to opt for less-than-ideal milk substitutes for their child.

Consistent with regional research and our experience with this population^5 21 22^ that identified various individual and systemic barriers to EBF, including food insecurity, insufficient milk supply and inadequate social support, our findings indicate that poor growth outcomes by 6 months of age are linked to early initiation of complementary feeding. Interventions in Pakistan to improve maternal knowledge of prelacteal feeding and EBF have shown modest increases in knowledge,^23 24^ but the factor of parental knowledge of EBF and IYCF alone may be insufficient in explaining poor child growth outcomes, since the prevalence of EBF practice remains low in resource-constrained settings such as Pakistan.^25 26^ In cases where parents notice their child’s failure to thrive, they will resort to alternative means of feeding their infant, which suggests that parental access to substitutes may be more significant than parental knowledge of EBF/IYCF. This suggests that addressing systemic barriers on multiple levels is essential, and individual-level counselling is but one solution for feeding practices. While interventions to improve IYCF knowledge are valuable, their effectiveness might be limited without addressing broader challenges related to sociocultural practices, maternal health and nutrition, paternal involvement and social attitudes, and women’s employment.^27^ Viable options tailored to the sociocultural and economic context are required. The finding that infants who are introduced to complementary feeding before 4 months of age have four times higher likelihood of wasting may not be reflective of the failure of parents. Rather, in our opinion and based on our experience,^5^ it indicates systemic issues within the healthcare sector and failure of healthcare leadership who should be providing contextually relevant support and guidelines for mothers who are not able to exclusively breastfeed, or afford formula, in resource-constrained settings such as Pakistan. Still, further specific research is required to differentiate between whether a child becomes malnourished after parents stop EBF, or if parents initiate complementary feeding earlier than recommended when perceiving inadequate infant growth from EBF.

One of the strengths of this study lies in its longitudinal design, with monthly follow-ups in a rural community during the first 6 months of infant development, allowing us to capture changes over time and identify associations between early feeding practices and subsequent growth outcomes. Parental recall bias and self-reporting of feeding practices are possible limitations, which we aimed to mitigate with monthly follow-up data collection visits by trusted community health workers. The significant sample size attrition (over 50%) of the birth cohort by 6 months of age was a notable study limitation. Furthermore, additional factors affecting feeding practices, such as maternal age, quality of supplemented diet and comorbidities, were not studied in the context of wasting/underweight status, due to which causal relations cannot be established in this study and which require examination in further studies.

Barriers to EBF in the region and similar resource-constrained settings have been identified.^10 12 27^ Future research should focus on developing feasible solutions to mitigate the associated risks of not being able to exclusively breastfeed in the first 6 months. Based on our study’s findings, we recommend further examination of the time to development of malnutrition and associated feeding practices before integrating individual-level interventions with additional system-level efforts to understand the reasons behind non-EBF and developing context-specific solutions to early on empower mothers and support healthy infant feeding practices when EBF is not feasible/possible.

### Conclusion

This study highlights the strong association between early complementary feeding initiation and the likelihood of infant wasting. Our study is comparable to existing qualitative literature on barriers to EBF, indicating that children may be wasting or underweight at birth or shortly after birth, due to which parents may resort to suboptimal feeding practices such as early initiation of complementary feeding. We emphasise the need for multifaceted interventions that address systemic challenges in resource-constrained settings, such as rural Pakistan.

## Supplementary material

10.1136/bmjph-2024-001204 online supplemental file 1

## Data Availability

All data relevant to the study are included in the article or uploaded as supplementary information.
