# Supplementary material for: Association of feeding practices with growth in infants: a longitudinal observational study in a rural district of Pakistan
Source: BMJ Public Health. 2025 Mar 28;3(1):e001204. doi: 10.1136/bmjph-2024-001204 (PMC11956284; doi:10.1136/bmjph-2024-001204)
Supplement: online supplemental file 1 [file bmjph-3-1-s001.pdf]

Supplementary Table 1: Associations between milk categories and malnutrition status

	**Wasting**	**Otherwise**	**Total**	**P-value**
	N=280	N=1,657	N=1,937	
**At 3 months**				0.010
Exclusive breastfeeding	30 (10.7%)	232 (14.0%)	262 (13.5%)	
Predominant BF	152 (54.3%)	990 (59.8%)	1,142 (59.0%)	
BF & formula milk	30 (10.7%)	93 (5.6%)	123 (6.4%)	
BF & animal milk	63 (22.5%)	323 (19.5%)	386 (19.9%)	
BF & both milk	1 (0.4%)	4 (0.2%)	5 (0.3%)	
Not breastfed	4 (1.4%)	14 (0.8%)	18 (0.9%)	
	N=30	N=1,245	N=1,275	
**At 6 months**				0.38
Exclusive breastfeeding	4 (13.3%)	78 (6.3%)	82 (6.5%)	
Predominant BF	13 (43.3%)	732 (59.3%)	745 (58.9%)	
BF & formula milk	2 (6.7%)	50 (4.0%)	52 (4.1%)	
BF & animal milk	11 (36.7%)	349 (28.3%)	360 (28.5%)	
BF & both milk	0 (0.0%)	10 (0.8%)	10 (0.8%)	
Not breastfed	0 (0.0%)	16 (1.3%)	16 (1.3%)	
	**Under-weight**	**Otherwise**	**Total**	**P-value**
	N=854	N=1,071	N=1,925	
**At 3 months**				0.008
Exclusive breastfeeding	102 (11.9%)	160 (15.0%)	262 (13.6%)	
Predominant BF	492 (57.6%)	644 (60.2%)	1,136 (59.0%)	
BF & formula milk	71 (8.3%)	49 (4.6%)	120 (6.2%)	
BF & animal milk	177 (20.7%)	206 (19.3%)	383 (19.9%)	
BF & both milk	3 (0.4%)	2 (0.2%)	5 (0.3%)	
Not breastfed	9 (1.1%)	9 (0.8%)	18 (0.9%)	
	N=485	N=787	N=1,272	
**At 6 months**				0.022
Exclusive breastfeeding	41 (8.5%)	41 (5.3%)	82 (6.5%)	
Predominant BF	276 (57.1%)	468 (60.1%)	744 (59.0%)	
BF & formula milk	23 (4.8%)	29 (3.7%)	52 (4.1%)	
BF & animal milk	127 (26.3%)	231 (29.7%)	358 (28.4%)	
BF & both milk	6 (1.2%)	4 (0.5%)	10 (0.8%)	
Not breastfed	10 (2.1%)	6 (0.8%)	16 (1.3%)	
